# Direct antigen presentation is the canonical pathway of cytomegalovirus CD8 T-cell priming regulated by balanced immune evasion ensuring a strong antiviral response

**DOI:** 10.3389/fimmu.2023.1272166

**Published:** 2023-12-12

**Authors:** Julia K. Büttner, Sara Becker, Annette Fink, Melanie M. Brinkmann, Rafaela Holtappels, Matthias J. Reddehase, Niels A. Lemmermann

**Affiliations:** ^1^ Institute for Virology and Research Center for Immunotherapy (FZI) at the University Medical Center of the Johannes Gutenberg University Mainz, Mainz, Germany; ^2^ Institute of Virology, Medical Faculty, University of Bonn, Bonn, Germany; ^3^ Institute of Genetics, Technische Universität Braunschweig, Braunschweig, Germany; ^4^ Virology and Innate Immunity Research Group, Helmholtz Centre for Infection Research, Braunschweig, Germany

**Keywords:** CD8 T cell response, m152/gp40, antigen presentation, antigen cross-presentation, antigen presenting cell (APC), murine cytomegalovirus (mCMV)

## Abstract

CD8 T cells are important antiviral effectors in the adaptive immune response to cytomegaloviruses (CMV). Naïve CD8 T cells can be primed by professional antigen-presenting cells (pAPCs) alternatively by “direct antigen presentation” or “antigen cross-presentation”. In the case of direct antigen presentation, viral proteins are expressed in infected pAPCs and enter the classical MHC class-I (MHC-I) pathway of antigen processing and presentation of antigenic peptides. In the alternative pathway of antigen cross-presentation, viral antigenic material derived from infected cells of principally any cell type is taken up by uninfected pAPCs and eventually also fed into the MHC class-I pathway. A fundamental difference, which can be used to distinguish between these two mechanisms, is the fact that viral immune evasion proteins that interfere with the cell surface trafficking of peptide-loaded MHC-I (pMHC-I) complexes are absent in cross-presenting uninfected pAPCs. Murine cytomegalovirus (mCMV) models designed to disrupt either of the two presentation pathways revealed that both are possible in principle and can substitute each other. Overall, however, the majority of evidence has led to current opinion favoring cross-presentation as the canonical pathway. To study priming in the normal host genetically competent in both antigen presentation pathways, we took the novel approach of enhancing or inhibiting direct antigen presentation by using recombinant viruses lacking or overexpressing a key mCMV immune evasion protein. Against any prediction, the strongest CD8 T-cell response was elicited under the condition of intermediate direct antigen presentation, as it exists for wild-type virus, whereas the extremes of enhanced or inhibited direct antigen presentation resulted in an identical and weaker response. Our findings are explained by direct antigen presentation combined with a negative feedback regulation exerted by the newly primed antiviral effector CD8 T cells. This insight sheds a completely new light on the acquisition of viral immune evasion genes during virus-host co-evolution.

## Introduction

Cytomegaloviruses (CMVs) belong to the β-subfamily of the herpesviruses [reviewed in (1)]. The medical relevance of human CMV (hCMV) is based on its pathogenicity and the resulting multiple organ CMV disease in the absence of immune protection. Risk is associated with congenital infection of the fetus and infection of immunocompromised patients with genetic or acquired immunodeficiencies. Patients with hematopoietic malignancies who undergo hematoablative therapy with subsequent hematopoietic cell transplantation (HCT) are at risk of developing symptomatic manifestations in the period before full reconstitution. In the case of allogeneic HCT, the risk is further enhanced by immunosuppressive therapy aimed at preventing graft-versus-host disease (GvHD). Likewise, CMV disease poses a threat to recipients of allogeneic solid organ transplantation (SOT) immunosuppressed for preventing graft rejection [reviewed in (2, 3)].

The mild and mostly unnoticed infection in the immunocompetent host reflects a largely reduced viral pathogenicity due to the control of viral replication by antiviral effector mechanisms of innate and adaptive immunity. Particularly during transient immunodeficiency in HCT patients, efficient reconstitution of antiviral CD8 T cells is associated with positive prognosis in both clinical CMV infection (4) and in experimental models [(5), reviewed in (6)]. Accordingly, the adoptive transfer of antiviral CD8 T cells is a promising immunotherapeutic approach to prevent CMV pneumonia and other organ manifestations of CMV infection in HCT recipients (3, 7–11).

Due to the strict host-specific replication of CMVs, hCMV cannot be studied in animal models (12, 13). As a versatile model system for natural host-virus pairs, the infection of mice with murine CMV (mCMV) has been established by many groups for investigating basic principles of viral pathogenesis and antiviral immune control (14–24). These principles are shared between different pairs of CMVs and their respective hosts, as coevolution has led to biological convergences in host-virus adaptation [reviewed in (9)].

For both hCMV and mCMV, CD8 T cells have been identified as major effectors in preventing viral pathology during acute infection (25, 26). In addition, the importance of CD8 T cells in the long-term surveillance of latent mCMV infection (27) is suggested by the expansion of certain viral epitope-specific populations of activated KLRG1^high^ CD62L^low^ CD8 T cells over time, a phenomenon termed “memory inflation (MI)” [for reviews, see (28–31)], and by an enhanced viral transcriptional activity during latency in the absence of such “inflating” CD8 T cells (32). Surprisingly, given the importance of CD8 T cells in the immune control of CMV and the interest in how MI is induced and maintained, the mechanisms underlying CMV-specific priming of naïve CD8 T cells are still not fully understood and remain controversial.

Viral antigenic peptides are presented, bound to MHC class-I (MHC-I) molecules as pMHC-I complexes, by professional antigen-presenting cells (pAPCs) to naïve CD8 T cells by two different mechanisms, direct presentation and cross-presentation. In the case of direct presentation, endogenous viral proteins are processed in infected pAPCs, whereas in the case of cross-presentation uninfected pAPCs take up exogenous antigens and introduce them into the MHC-I pathway of antigen processing and presentation (33). As a potential source of direct antigen presentation, mCMV can infect pAPCs, such as dendritic cells (DCs) (34, 35) and CD169^+^ macrophages (36).

However, all CMVs code for proteins that manipulate the MHC-I pathway of antigen presentation and are known as viral regulators of antigen presentation (vRAP) [(35), for more recent reviews see (19, 37)]. For mCMV, three vRAPs have been described: the positive regulator m04/gp34 and the negative regulators m06/gp48 and m152/gp40. Recent work has shown that m04 and m06, which belong to the same protein family (37), compete for pMHC-I cargo and annihilate each other in their function (38). For this reason, m152 remains as the functionally relevant immunoevasin of mCMV (38) that traps pMHC-I complexes in a cis-Golgi compartment (39–42), thereby reducing their number available for interaction with CD8 T-cell receptors (TCRs) (43). Notably, recent work has shown that a reduction of the number of cell surface pMHC-I molecules raises the avidity threshold required for TCRs of antiviral CD8 T cells to recognize infected cells and protect against infection [reviewed in (44)]. As a consequence, the recognition of infected cells by virus-specific CD8 T cells is impaired by the action of m152 *in vitro* and *in vivo* (35, 45, 46). Besides downmodulating pMHC-I, m152 has also been shown to interfere with cell surface expression of RAE-1 family ligands of the activating NK-cell receptor NKG2D (47–49), thereby preventing NK-cell activation (50, 51). Furthermore, m152 targets STING to reduce induction of type I antiviral interferons (52). This multi-functionality indicates a key role for m152 in subverting the antiviral defense.

Since vRAPs have been shown to be functional in pAPCs after hCMV and mCMV infection (34, 35, 53–55), it has been hypothesized that cross-presentation is the major pathway of antigen presentation leading to effective CD8 T-cell priming by counteracting viral immune evasion (56–58). Consistent with this, MHC-I-negative fibroblasts infected with a spread-deficient mCMV, which prevents direct presentation in the first round and precludes further rounds of infection, have been shown to activate mCMV-specific CD8 T cells after immunization of WT mice (59). Furthermore, Batf3^-/-^ mice, which lack cross-presenting CD8α^+^ and CD107^+^ DCs, showed impaired priming (60). All these results clearly indicate that, in principle, cross-presentation can occur during mCMV infection.

On the other hand, there is evidence that priming by cross-presentation does not exclude a role for direct presentation. While Batf3^-/-^ mice completely lack CD8^+^ DCs, which could contribute also to direct priming, infection of CD11c-Rac mice, which are selectively defective in the uptake of exogenous antigens by DCs, showed an mCMV-specific priming comparable to that in WT mice (57). In analogy, mCMV-specific priming was not impaired in mice treated with the TLR9 agonist CpG to prevent cross-presentation (61). In addition, there is evidence that the downregulation of pMHC-I is less efficient in DCs and macrophages than it is in fibroblasts or endothelial cells (62–64). Overall, it remained controversial whether direct or cross-presentation is the canonical pathway for the induction of CD8 T-cell responses to CMV.

Previous approaches are characterized by blocking either direct antigen presentation or cross-presentation, and thus were, by concept, unable to decide which pathway is taken with preference in normal mice. Here we present a novel approach in which priming of CD8 T cells is studied under conditions of enhanced or reduced immune evasion, compared to infection with WT virus, by using recombinant viruses in which the key immune evasion protein m152 is overexpressed or deleted, respectively. If priming is achieved by direct antigen presentation, the CD8 T-cell response is expected to be reduced after enhanced immune evasion and increased after reduced immune evasion. Surprisingly, our data on CD8 T-cell priming in a regional lymph node (RLN) did not reveal such a difference. Rather, up- or down-modulation of immune evasion gene expression led to the same response magnitude in the net effect. It thus appeared more than logical to conclude that priming is not by direct antigen presentation, thereby providing indirect evidence and an argument for priming by cross-presentation. However, this tempting conclusion ignores the fact that the modulation of direct antigen presentation has an influence on the recognition of infected cells by the newly primed CD8 T cells. In a negative feedback loop, a high number of CD8 T cells is efficiently primed by enhanced direct antigen presentation limiting viral replication and thus the number of APCs available for further rounds of T-cell stimulation. In contrast, a low number of CD8 T cells generated initially after reduced direct antigen presentation inefficiently limits viral replication and thus leads to a higher number of infected APCs driving further rounds of antigen presentation. From this “immune evasion paradox”, we conclude that direct antigen presentation is the canonical pathway of mCMV-specific CD8 T-cell priming within RLNs.

## Materials and methods

### Cells, viruses, and mice

P815 (No. TIB-64, haplotype H-2^d^) and EL4 (No. TIB-39, haplotype H-2^b^) cells were obtained from the American Tissue Culture Collection (ATCC) and cultivated in RPMI supplemented with 5% fetal calf serum (FCS) and antibiotics, or in DMEM with 10% FCS and antibiotics, respectively. Primary murine embryo fibroblasts (MEF) were cultivated in MEM supplemented with 10% FCS and antibiotics. A CD8 T-lymphocyte line (CTLL) specific for the immunodominant viral epitope IE1 (YPHFMPTNL) (65) was generated from spleen-derived memory CD8 T cells of latently infected BALB/c mice by four rounds of restimulation with synthetic peptide (66).

Virus derived from BAC plasmid pSM3fr (67) was used as “wild-type” (WT) virus, mCMV-WT. Recombinant viruses mCMV-Δm152 (40), mCMV-Δm157 (68), and mCMV-m152StopΔm157 (52) have been described previously.

BALB/c, C57BL/6, and C57BL/6-Unc93b1^3d/3d^ [briefly Unc93b1^3d/3d^ (69)] mice were bred and housed under specified-pathogen-free (SPF) conditions in the Translational Animal Research Center (TARC) at the University Medical Center of the Johannes Gutenberg-University Mainz, Germany, and at the central animal facility of HZI Braunschweig, Germany.

### Generation of recombinant virus

Recombinant plasmids were constructed according to established procedures, and enzyme reactions were performed as recommended by the manufacturers. Throughout, the fidelity of PCR-based cloning steps was verified by sequencing (GATC, Freiburg, Germany).

Mutagenesis of full-length mCMV BAC plasmid pSM3fr was performed in DH10B by using the two-step replacement method as described (67, 70), resulting in the BAC plasmid pSM3fr_m152.IE+E. For this, the shuttle plasmid pST76K_ie2_m152 was used to integrate the open reading frame (ORF) m152 for ectopic expression under the control of the ie2 promoter. In the first step, the intermediate plasmid pST76K_ie1/3-ie2 was generated by subcloning a *Pml*I-cleaved 5,557bp fragment of pUCAMB (71), containing nucleotides 181,415 to 186,972 (GenBank accession no. NC_004065) of the mCMV immediate-early (IE) region into the *Sma*I site of the shuttle plasmid pST76-KSR (70). In a subsequent step, a 1,452bp PCR fragment, encompassing the ie2 promoter and ORF m152, was introduced into the *Hpa*I cleaved vector to generate pST76K_ie2_m152. The fragment was generated by a touchdown PCR with primer pair m152-*Hpa*I-fwd GAA**GTTAAC**_184,240_CATATAAAAGCTGTCCCCCATGCCATTCGA_184,269-211,468_TCAGACGCGGGCTACTCCCGAAAGAGTAAC_211,439_ and m152-*Hpa*I-rev GGA**GTTAAC**_210,056_TGACTAATAAGTTATCTTTATTGTACAAGTGTTGTGTGTTATCCCTGAGCCCATTCCCAG_210,115_ (*Hpa*I restriction sites are indicated in bold letters) using ProofStart Taq DNA polymerase (catalog no. 202205; QIAGEN, Hilden, Germany) and cycler conditions as follows: an initial step for 5 min at 95°C was followed by 18 cycles for 30 s at 94°C, 120 s with temperatures decreasing by 1°C per cycle starting from 62°C, and 90 s at 68°C each, followed by 12 cycles for 30 s at 94°C, 120 s at 45°C, and 90 s at 68°C.

Reconstitution and purification of a high-titer virus stock of mCMV-m152.IE+E was performed as described (72).

### Infection conditions and virus growth kinetics in immunocompromised mice

For *in vitro* assays, MEF were infected with the indicated viruses at a multiplicity of infection (MOI) of 4 with centrifugal enhancement of infectivity (72–74). Intraplantar infection of 8-to-10-week-old mice was performed in the left hind footpad with 1x10^5^ PFU of the respective virus.

For log-linear *in vivo* virus growth curves, BALB/c mice were immunocompromised by hematoablative treatment with a single 6.5 Gy dose of total-body γ-irradiation and were infected with the respective virus. Quantification of viral genome load in lungs and spleen was performed on days 2, 4, 6, 8, and 10 by qPCR as described previously (72).

### Depletion of lymphocyte subsets *in vivo*

Depletion of NK cells or of CD8 T cells was performed 24 h prior to infection by i.v. injection of 25µl rabbit antiserum directed against asialo-GM1 (catalog no. 986-100001; Wako Chemicals, Osaka, Japan) or of 1mg purified antibody directed against CD8 (clone YTS169.4), respectively (75).

### Quantification of viral genomes and transcripts

To determine viral genome load in lungs and spleen, DNA of infected mice was isolated from the respective tissues with the DNeasy blood & tissue kit (catalog no. 69504; QIAGEN) according to the manufacturer’s instructions. Viral and cellular genomes were quantitated in absolute numbers by M55-specific and pthrp-specific qPCRs normalized to a log_10_-titration of standard plasmid pDrive_gB_PTHrP_Tdy (72, 76).

Viral transcripts were quantitated from total RNA extracted from infected MEF or from lymph nodes (75), and 500 ng RNA was used as template for RT-qPCR. Absolute quantification of E1 or m152 transcripts using *in vitro* transcripts as standard has been described previously (77). Spliced E1 transcripts (78, 79) were chosen as a surrogate for viral replication that otherwise would be confounded by inoculum viral DNA. It is important to recall that a PFU of mCMV equals 500 copies of viral genomic DNA (73), so that intraplantar infection with 1x10^5^ PFU corresponds to 5x10^7^ copies, which critically confounds the quantitation of *de novo* viral DNA replication in the RLN, in particular at early times. E1 (M112-113) expression is essential for viral DNA replication (80) and the quantity of E1 transcripts correlates with the number of infected cells.

### Peptides

Custom peptide synthesis to a purity of > 80% was performed by JPT Peptide Technologies (Berlin, Germany). Synthetic peptides representing antigenic peptides in mouse haplotype H-2^b^ were M38 (SSPPMFRVP), M45 (HGIRNASFI), M57 (SCLEFWQRV), M122/IE3 (RALEYKNL), m139 (TVYGFCLL), and m141 (VIDAFSRL) (81). Those for mouse haplotype H-2^d^ were m04 (YPGSLYRRF), m18 (SGPSRGRII), M45 (VGPALGRGL), M83 (YPSKEPFNF), M84 (AYAGLFTPL), M105 (TYWPVVSDI), m123/IE1 (YPHFMPTNL), m145 (CYYASRTKL), and m164 (AGPPRYSRI) (8). The synthetic peptides were used for exogenous loading of target cells in the ELISpot assay.

### ELISpot assay

An interferon gamma (IFNγ) enzyme-linked immunospot (ELISpot) assay was performed for quantification of IFNγ-secreting CD8 T cells after sensitization by peptide-loaded stimulator cells. Frequencies of mCMV-specific CD8 T cells were determined by incubation of graded numbers of immunomagnetically-purified CD8 T cells, derived from the RLN, which is the popliteal lymph node in the case of intraplantar infection, with stimulator cells (P815 or EL4, as it applied) that were exogenously loaded with synthetic peptides at a saturating concentration of 10^-7^M (75). IE1 epitope presentation after endogenous antigen processing in infected BALB/c MEF was determined using short-term IE1-CTLL (82) as responder cells. Spots were counted automatically based on standardized criteria using Immunospot S4 Pro Analyzer (CTL, Shaker Heights, OH, USA) and CTL-Immunospot software V5.1.36. Frequencies of IFNγ-secreting cells and the corresponding 95% confidence intervals were calculated by intercept-free linear regression using Mathematica, version 8.0.4.

### IE phase arrest of infected cells

For a selective arrest of viral gene expression in the IE phase, MEFs were infected in the presence of 50µg/ml cycloheximide (CHX) to block protein synthesis reversibly. At 3 h after infection, the culture medium was replaced by fresh medium containing 5 µg/µl actinomycin D (ActD) as described previously (83).

### Intracellular cytokine assay

IE1-CTLL (5x10^5^ cells) were co-cultivated with 1x10^5^ mCMV-infected, IE phase-arrested MEFs (BALB/c, haplotype H-2^d^) for 5h at 37°C in the presence of brefeldin A (BD GolgiPlug, final concentration 1:1,000; catalog no. 555029; BD Biosciences). Thereafter, the CTLL cells were fixed, permeabilized with BD Cytofix/Cytoperm (catalog no. 554722, BD Biosciences) and stained with FITC-conjugated anti-mouse IFNy antibody (clone XMG1.2, catalog no. 554411, BD Biosciences) for cytofluorometric (CFM) analysis performed with flow cytometer Cytomics FC500 and CXP analysis software (Beckman Coulter).

### Genome-wide ORF library screening

An mCMV ORF library of expression plasmids spanning the entire mCMV genome (81) was used for ORF-specific stimulation of *ex vivo* isolated CD8 T cells with transfected SV-40 fibroblasts, followed by CFM detection of intracellular IFNγ.

### Immunoblot analysis

The expression of mCMV proteins was detected by Western blot analysis as described (40). In brief, lysates of infected MEF were prepared and 30µg of total protein amount was subjected to separation by 12.5% SDS-PAGE, followed by blotting onto polyvinylidene difluoride membrane and protein labeling with the respective antibodies. The following antibodies were used: m152 (M3D10, monoclonal antibody, kindly provided by E. Kremmer, Helmholtz Zentrum München, Munich, Germany), IE1 (Croma 101, monoclonal antibody, kindly provided by S. Jonjic, University of Rijeka, Rijeka, Croatia), and IE2 (αIE2-N, polyclonal antibody, rabbit, Peptide Specialty Laboratories, Heidelberg, Germany). Detection of antibody binding was visualized by chemiluminescence using the ECLplus Western blotting detection system (catalog no. RPN2132; Amersham Bioscience, Little Chalfont, United Kingdom) and Lumi-Film (catalog no. 11666657001, Roche Applied Science, Mannheim, Germany).

### Statistical analysis

To evaluate statistical significance of differences between two independent sets of data, the unpaired t-test with Welch’s correction of unequal variances was used. Differences are considered statistically significant for P values <0.05 and highly significant for P values <0.001. In cohort analyses of viral epitope-specific CD8 T cells by the ELISpot assay, differences are considered statistically significant if 95% confidence intervals do not overlap.

Virus doubling times (vDT) and their 95% confidence intervals (CI) were calculated from the slopes of log-linear regression lines determined by linear regression analysis (84–86). Calculations were performed with Graph Pad Prism 10 (Graph Pad Software, San Diego, CA). It should be noted that vDT values vary between different organs but are a constant for each organ independent of the viral replication parameter tested, that is, identical for viral genomic DNA copy numbers measured by qPCR, infectious virus expressed as PFU, or numbers of infected tissue cells determined by immunohistological detection of viral proteins (85).

## Results

### Broad CD8 T-cell response to mCMV in C57BL/6 mice genetically deficient in the antigen cross-presentation pathway

Conclusions on the mechanism of CD8 T-cell priming are usually based on measuring the magnitude of the primary immune response. This includes the tacit assumption that antigen presentation requirements are identical for sensitization of naïve CD8 T cells by antigen presentation in the immunological synapse (87, 88), which is the initial priming event that requires pAPCs, and for subsequent clonal expansion. Accordingly, the terms of “priming” and “primary response” are usually used as synonyms, not least because the initial priming event is difficult to access. Thus, while a response indicates a successful initial priming, one must keep in mind that one actually looks at the combined result of priming and subsequent clonal expansion.

To provide evidence for or against either of the two pathways of antigen presentation, we studied here the mCMV-specific CD8 T-cell response in an RLN draining a site of local infection, specifically in the popliteal lymph node after intraplantar infection. The RLN is the anatomical structure where direct priming is said to occur in the peripheral interfollicular region (89–91). Our work builds on a previous study (75) in which we have shown that mCMV rapidly reaches the RLN, infects cells in the peri-subcapsular sinus region, and generates virus-specific effector CD8 T cells as early as by day 3 after virus exposure. Notably, this finding is in perfect accordance with the more recent study by Reynoso and colleagues (92) showing that lymph node conduits rapidly transport virions to infect pAPCs in the RLN paracortex followed by rapid direct T-cell priming within the T-cell zone.

**Figure 1:**
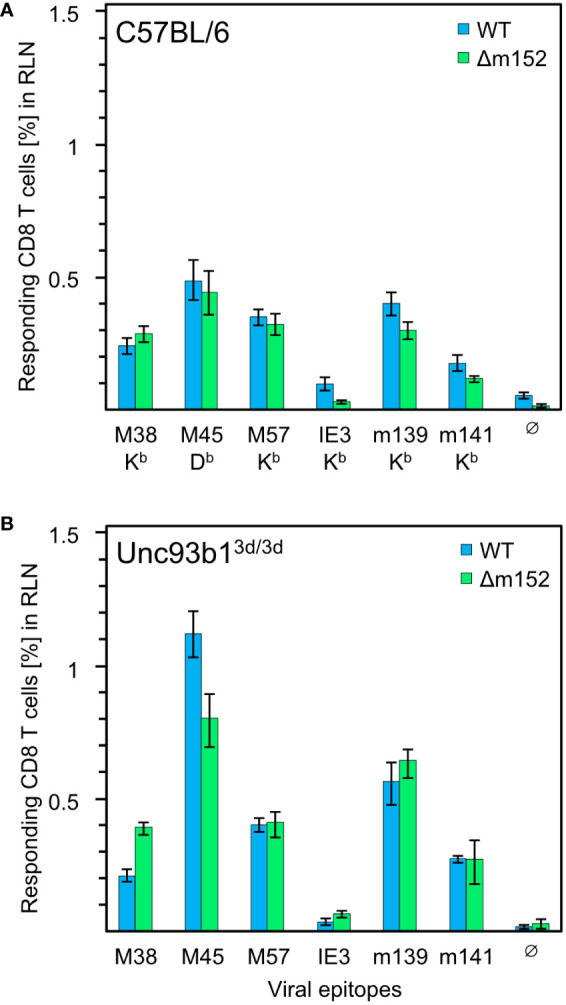
CD8 T-cell response induced by mCMV in presence and absence of the cross-presentation pathway. **(A)** CD8 T-cell response in C57BL/6 mice (n=5 per group/virus, tested as cohorts). **(B)** CD8 T-cell response in Unc93b1^3d/3d^ mice (n=4 per group/virus, tested as cohorts) that are deficient in antigen cross-presentation. CD8 T cells were isolated from the draining regional lymph node (RLN), that is, the popliteal lymph node, on day 7 after intraplantar infection with 1x10^5^ PFU each of either mCMV-WT (WT) or mCMV-Δm152 (Δm152), and used as effector cells in an IFNγ-based ELISpot assay. EL4 cells exogenously loaded with synthetic peptides at a saturating concentration of 10^−7^ M were used as APCs. The panel of tested antigenic peptides and the corresponding peptide-presenting MHC-I molecules are indicated. Bars represent cohort average most probable numbers (MPN) of responding cells determined by intercept-free linear regression analysis, and error bars represent the corresponding 95% confidence intervals. Test groups are considered significantly different if the 95% confidence intervals do not overlap. Ø, no peptide added.

In the specific case of CMV infections, however, viral interference with the MHC-I pathway of direct antigen presentation was expected to prevent or at least severely inhibit CD8 T-cell priming. To test this prediction, we analyzed the mCMV-specific CD8 T-cell response in Unc93b1^3d/3d^ mice. This strain on C57BL/6 genetic background is known to lack endosomal TLR3, 7, and 9 signaling and is impaired in exogenous antigen processing, resulting in a blockade of cross-presentation (69, 93–96). Accordingly, CD8 T-cell priming in these mice ought to largely depend on direct presentation. Previous reports on mCMV infection in this mutant mouse strain revealed an impaired cytokine production but comparable frequencies of M45-specific hepatic CD8 T cells (69, 97). Herein, we compared the CD8 T-cell response in C57BL/6 WT and Unc93b1^3d/3d^ mice not just for the M45 epitope but for a panel of known mCMV peptides presented in the H2^b^ haplotype (**Figure 1**).

Overall, no qualitative differences in the immunodominance patterns were found. To our surprise, the magnitude of the CD8 T-cell response to some of the peptides tested, most markedly for M45, was even slightly higher in Unc93b1^3d/3d^ mice, but certainly not lower. This finding is consistent with data reported for C57BL/6 mice in which cross-presentation was suppressed by CpG pretreatment (61). In summary, the magnitude of the primary CD8 T-cell response is surprisingly not at all inhibited by viral interference with direct antigen presentation, which is known to be effective at the cellular level following infection with mCMV-WT in several cell types tested, including pAPCs, and in mice of haplotypes H-2^b^ and H-2^d^ (35, 98).

Given the remarkable finding that a CD8 T-cell response occurs despite genetic prevention of cross-presentation and despite viral interference with direct antigen presentation, we wondered whether the magnitude of the response would at least benefit from improved direct antigen presentation. For this, we compared infection with mCMV-WT and the immune evasion gene deletion mutant mCMV-Δm152 both under conditions with an open (**Figure 1A**) or closed (**Figure 1B**) antigen cross-presentation pathway in C57BL/6 and Unc93b1^3d/3d^ mice, respectively. The result was most striking: for the entire panel of viral epitopes tested, improved direct antigen presentation failed to improve the response, regardless of whether the antigen cross-presentation pathway was accessible or not. For C57BL/6 mice, earlier work by the group of A.B. Hill (98, 99) has already shown that an enhancement of direct antigen presentation by deletion of the viral key immune evasion gene m152 has little impact on the magnitude of the CD8 T-cell response, having suggested that priming may rather depend on antigen cross-presentation. Based on the data in Unc93b1^3d/3d^ mice, however, this explanation has now become obsolete, and we are faced with the riddle that the CD8 T-cell response in mice with the genetic background of C57BL/6 is resilient and buffered in that it neither depends on antigen cross-presentation nor does it appear to be notably influenced by enhanced direct antigen presentation.

### An early NK-cell response simultaneously restricts intranodal viral replication and the CD8 T-cell response in C57BL/6 mice

Selectively in mice with the genetic background of C57BL/6, the viral protein m157 restricts viral replication by serving as an activatory ligand of Ly49H^+^ NK cells (100, 101). It has been shown that the activation of Ly49H^+^ NK cells by m157 suppresses the mCMV-specific CD8 T-cell response, most likely by reducing the number of infected pAPCs available for direct antigen presentation (102). Thus, Ly49H^+^ NK cells are a relevant player that certainly contributes to the magnitude of the CD8 T-cell response in C57BL/6 mice.

**Figure 2:**
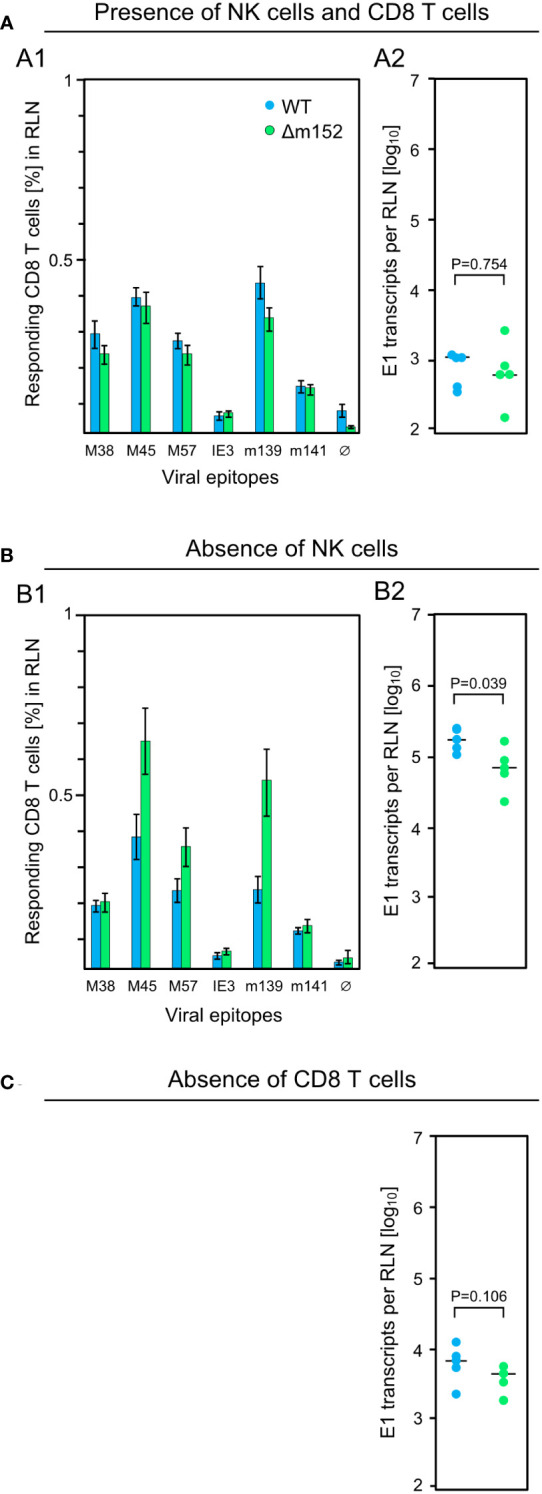
Influence of lymphocyte subsets on the CD8 T-cell response and the intranodal viral replication in C57BL/6 mice. Intraplantar infection of immunocompetent C57BL/6 mice (n=5 per group) was performed with 1x10^5^ PFU each of either mCMV-WT (WT) or mCMV-Δm152 (Δm152). **(A)** No depletion of lymphocyte subsets. **(B)** Depletion of NK cells one day before infection. **(C)** Depletion of CD8 T cells one day before infection. The CD8 T-cell response in the RLN on day 7 post-infection was assessed by an IFNγ-based ELISpot assay (A1, B1), as explained in greater detail in the legend of **Figure 1**. As a surrogate for viral replication in the presence of otherwise confounding viral inoculum DNA, spliced E1 transcripts present in the RLN were quantitated by RT-qPCR on day 3 post-infection (A2, B2, **C**). Symbols represent individual mice and horizontal bars indicate median values. P values were calculated based on the log-transformed data with Welch´s unpaired t-test (two-sided). Differences are considered significant for P < 0.05.

For clarification, we investigated the impact of NK cells on the CD8 T-cell response in the draining RLN measured on day 7 after intraplantar infection of C57BL/6 mice, and correlated the response magnitude with viral replication at this site on day 3 (**Figure 2**). In the presence of NK cells and CD8 T cells, mCMV-WT and mCMV-Δm152 induced similar CD8 T-cell responses (**Figure 2A1**), consistent with the preceding, independent experiment (**Figure 1A**). Notably, transcription of viral gene E1, which serves as a surrogate for viral replication, was identical for both viruses and on a low level (**Figure 2A2**). In contrast, pan-NK cell depletion prior to infection led to an increase in the response magnitude for the immunodominant viral peptides M45, M57, and m139, but only when direct antigen presentation was enhanced by deletion of m152 (**Figure 2B1**). As it was predicted, depletion of NK cells led to a greatly enhanced viral replication in the RLN (**Figure 2B2**) compared to the control group with no NK-cell depletion (**Figure 2A2**). Deletion of m152 resulted in a slight, but statistically significant, reduction in viral replication compared to WT virus infection (**Figure 2B2**). This reduction was caused by recently primed CD8 T cells, as the difference was abolished by the depletion of CD8 T cells (**Figure 2C**).

Upon first impression, it may be irritating that not all viral epitopes show the same response pattern. Epitope hierarchy, however, is a general observation and is explained by differences in many consecutive steps in the MHC-I pathway of antigen processing and presentation. Key variables, which are even used in epitope prediction algorithms, include the efficacy of peptide-generating proteasomal cleavage and peptide binding affinity to the presenting MHC-I molecule. Cell surface density of pMHC-I complexes determines the cooperative TCR binding avidity and thus the intensity and duration of TCR signaling. This in turn defines the proliferation rate of CD8 T cells for clonal expansion and thus, finally, the response magnitude. As a consequence, differences that do not reach statistical significance for subdominant viral epitopes can reach statistical significance for dominant viral epitopes. For this reason, we always tested a panel of epitopes, and conclusions must be drawn from the overall picture rather than from single epitopes. Our data did not reveal a critical influence of the type of the presenting MHC-I molecule, which are K^b^ and D^b^ in C57BL/6 mice.

**Figure 3:**
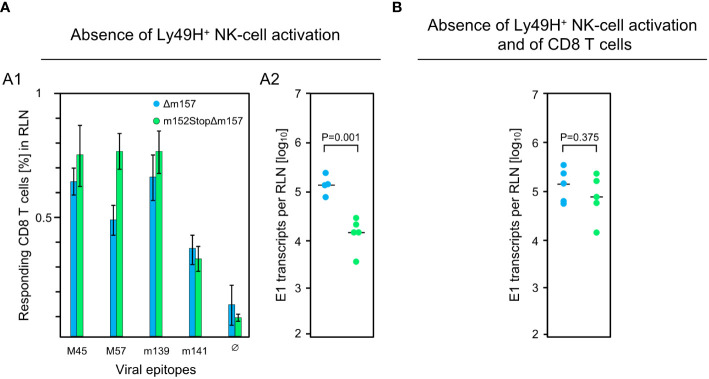
Impact of Ly49H^+^ NK cells on CD8 T-cell response and intranodal viral replication in C57BL/6 mice. **(A)** Absence of Ly49H^+^ NK-cell activation. **(B)** Absence of Ly49H^+^ NK-cell activation and of CD8 T cells. Intraplantar infection of immunocompetent C57BL/6 mice (n=5 per group) was performed with 1x10^5^ PFU each of either mCMV-Δm157 (Δm157) or mCMV-m152StopΔm157 (m152StopΔm157). (A1) The CD8 T-cell response in the RLN on day 7 post-infection was assessed by an IFNγ-based ELISpot assay, as explained in greater detail in the legend of **Figure 1**. (A2, **B**) Spliced E1 transcripts present in the RLN on day 3 post-infection were quantitated by RT-qPCR. Depletion of CD8 T cells in **(B)** was performed on the day before infection. Symbols represent data from individual mice and horizontal bars indicate median values. P values were calculated based on the log-transformed data with Welch´s unpaired t-test (two sided). Differences are considered significant for P < 0.05.

These findings were essentially reproduced in an independent experiment using the alternative approach of testing the influence of m152 on the response magnitude in infected C57BL/6 mice in the absence specifically of Ly49H^+^ NK cell activation via Ly49H-m157 ligation, instead of pan-NK cell depletion (**Figure 3**). For this, we compared response magnitude and viral replication in the RLN after infection with mCMV-Δm157, expressing m152, and mCMV-m152StopΔm157, lacking m152 expression, both in the absence of Ly49H^+^ NK cell activation. Notably, deletion of m152 increased the overall CD8 T-cell response (**Figure 3A1**), more or less paralleling the findings after pan-NK cell depletion (**Figure 2B**). Under these conditions, too, early recognition of infected cells is indicated by a reduction in viral replication in the RLN after infection with mCMV-m152StopΔm157 (**Figure 3A2**). Again, this antiviral control in the absence of Ly49H^+^ NK cell activation is mediated by recently primed CD8 T cells, as depletion of CD8 T cells led to an increase in viral replication in the RLN (**Figure 3B** compared to **Figure 3A2**).

The results of the two approaches are consistent in having demonstrated that early viral replication in the RLN of C57BL/6 mice is mainly controlled by Ly49H^+^ NK cells. While an enhanced direct antigen presentation did not improve the overall CD8 T-cell response magnitude in the presence of the NK-cell response (**Figure 2A1**), depletion or missing activation of NK cells disclosed an improvement (**Figures 2B1**, **3A1**). Importantly, a high CD8 T-cell response corresponded to low intranodal viral replication, and a low response corresponded to high viral replication (**Figures 2B**, **3A**). This clearly argues against antigen cross-presentation for which the opposite should apply: high virus replication and thus high amounts of antigenic proteins available for uptake by uninfected pAPCs ought to correspond to a high CD8 T-cell response and, accordingly, low virus replication ought to correspond to a low CD8 T-cell response. In summary, although masked by the strong activity of Ly49H^+^ NK cells, CD8 T cells in C57BL/6 mice are primed primarily by direct antigen presentation.

### Combined kinetic acceleration and enhancement of immune evasion strongly inhibit direct antigen presentation in infected cell culture

Since the mCMV-specific CD8 T-cell response in C57BL/6 mice is masked by the strong response of Ly49H^+^ NK cells, we decided to switch to the analysis of the mCMV-specific CD8 T-cell response in BALB/c mice, which do not express Ly49H and accordingly lack this functionally dominant subset of NK cells. Based on the evidence that the CD8 T-cell response in C57BL/6 mice is driven by direct antigen presentation (see above), and assuming that this is also the case in BALB/c mice, we reasoned that the best evidence for direct antigen presentation would be to show that enhanced and inhibited direct antigen presentation correspond to a high and low CD8 T-cell response, respectively. Increased direct antigen presentation compared to infection with the WT virus is achieved by deletion of the immune evasion gene m152 in virus mCMV-Δm152. For a more strongly inhibited direct antigen presentation compared to infection with the WT virus, we constructed a “super-evasion” virus mCMV-m152.IE+E as a new study tool.

In mCMV-WT infection, m152 is expressed quite early in the Early (E) phase of the viral replication cycle (24, 39). Antigens expressed even earlier, that is, in the Immediate-Early (IE) phase, may profit from a head start advantage of presentation before immune evasion can operate. After infection with mCMV-m152.IE+E, m152 is expressed from the IE phase onward.

**Figure 4:**
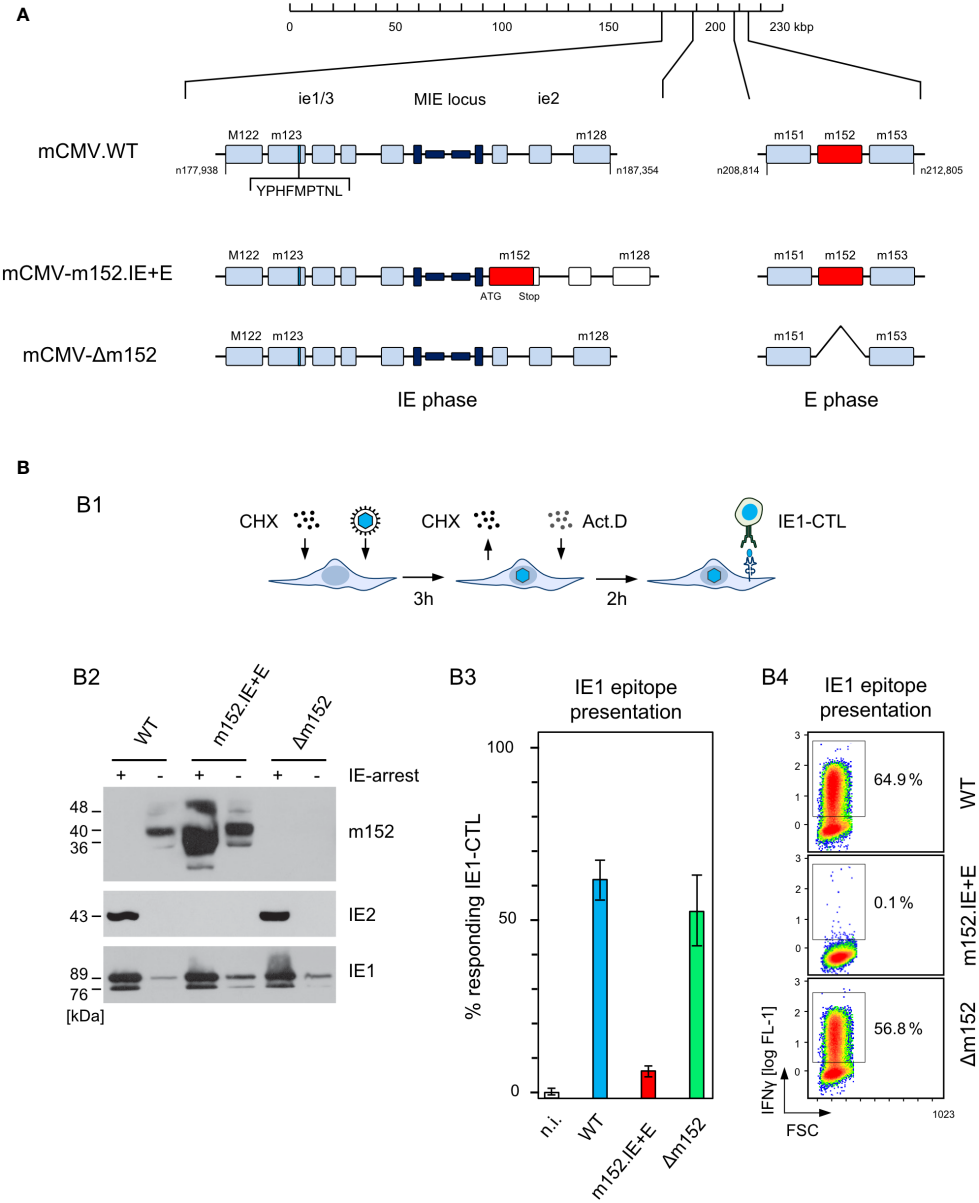
Construction and *in vitro* characterization of recombinant virus mCMV-m152.IE+E. **(A)** Maps, illustrating the mutagenesis design. A map of the mCMV genome is shown at the top of the sketch. The genomic region that includes the parental E phase gene m152 is shown expanded on the right-hand side. The “Major Immediately Early (MIE)” locus, consisting of the MIE promotor-enhancer-enhancer-promotor (dark blue) flanked by transcription units ie1/3 and ie2, is shown expanded on the left-hand side. Exons are indicated by boxes (light blue). The coding sequence of the IE1 peptide located in exon 4 of the ie1/3 transcription unit is marked. Recombinant virus mCMV-m152.IE+E, expressing m152 in both the IE phase and the E phase, was generated by introducing the ORF m152 by two-step BAC mutagenesis into the MIE locus under the control of the ie2 enhancer-promoter element, thereby disrupting the ie2 gene. **(B)** Immune evasion under selective IE phase conditions. (B1) Experimental protocol for arresting infection in the IE phase. BALB/c-derived mouse embryonal fibroblasts (MEF) were infected in the presence of cycloheximide (CHX) that was replaced at 3 h post-infection with actinomycin D (ActD). The thus IE phase-arrested MEF were harvested for analyses at 5 h post-infection. (B2) Western blot analysis of m152 (40kDa and additional glycosylation isoforms), IE2 (43kDa) and IE1 (89/76kDa) protein expression in IE phase-arrested (+) or untreated (–) MEF infected with the indicated viruses. (B3) IFNγ-based ELISpot analysis quantifying cells of an IE1-CTLL that were sensitized by IE phase-arrested BALB/c-derived MEF infected with the indicated viruses. Bars represent most probable numbers (MPN) of responding cells determined by intercept-free linear regression analysis, and error bars represent the corresponding 95% confidence intervals. Test groups are considered significantly different if the confidence intervals do not overlap. (n.i.) not infected. (B4) Intracellular IFNγ-staining of IE1-CTLL cells at 5 h after co-cultivation with IE phase-arrested MEF infected with the indicated viruses. Two-dimensional color-coded density plots (with red and blue representing highest and lowest density, respectively) show intracellular IFNγ expression (ordinate; fluorescence intensity) versus the forward scatter (abscissa; FSC, linear scale of channels) with 50,000 cells analyzed. The percentages of IFNγ^+^ cells present in the demarcated gates are indicated.

Ectopic expression in the IE phase was achieved by insertion mutagenesis placing ORF m152 under the control of the ie2 enhancer-promoter, thereby disrupting the ie2 gene. Expression as an E phase protein occurred from its authentic position in the mCMV genome (**Figure 4A**). For verifying immune evasion operating already in the IE phase, viral gene expression in BALB/c-derived fibroblasts was metabolically arrested in the IE phase (**Figure 4B1**). This led to an enhanced synthesis of IE proteins IE1 and IE2, and absence of the m152 protein, after infection with either mCMV-WT or the deletion mutant mCMV-Δm152, whereas all three glycosylation isoforms of m152 (40) were strongly expressed and IE2 was absent after infection with mCMV-m152.IE+E (**Figure 4B2**, ). Presentation of the antigenic peptide IE1 (YPHFMPTNL, presented by L^d^) was tested functionally with an IE1 epitope-specific CTLL (IE1-CTLL) used as responder cells in an IFNγ-based ELISpot assay with IE phase-arrested infected cells as stimulator cells (**Figure 4B3**) as well as by intracellular cytofluorometric staining of IFNγ in IE1-CTLL cells sensitized by IE phase-arrested infected cells (**Figure 4B4**). In both assays, IE1 peptide was presented without significant difference after infection with either mCMV-WT or the mCMV-Δm152 deletion mutant, since m152 is not expressed in the IE phase anyway. In contrast, presentation was blocked in IE phase-arrested cells after infection with mCMV-m152.IE+E (**Figures 4B3, B4**).

**Figure 5:**
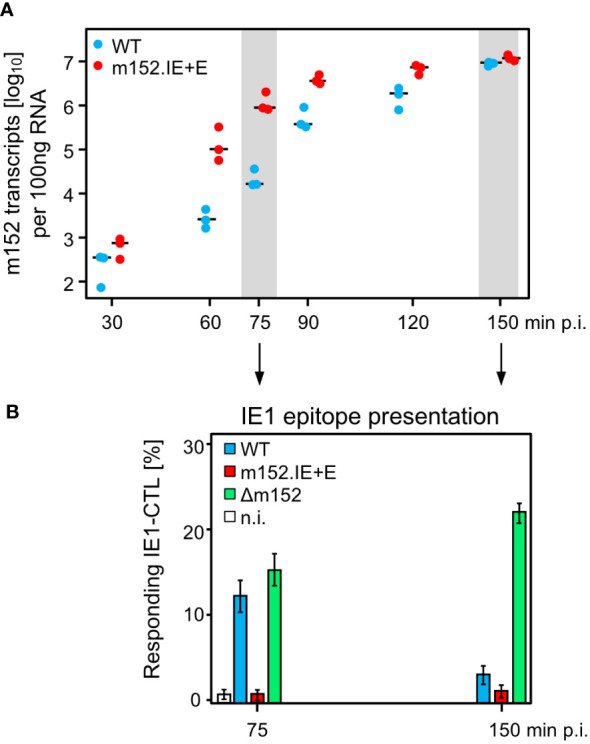
mCMV-m152.IE+E infection inhibits IE1 peptide presentation from the start of viral gene expression. **(A)** Quantitation of m152 transcripts in the time-course. BALB/c-derived MEF were infected with the indicated viruses and m152 transcripts were quantitated by RT-qPCR, normalized to 100 ng of total RNA, at the indicated times post-infection. Symbols represent biological replicates (n=3). Median values are indicated by horizontal bars. **(B)** IE1-peptide presentation at selected times. IE1-peptide presentation was detected at the indicated times based on sensitization of IE1-CTLL cells in an IFNγ-based ELISpot assay by BALB/c-derived MEF infected with the viruses indicated. To avoid ongoing transcription in the stimulator cells during the ELISpot assay time, the transcription inhibitor ActD was added at the indicated times of cell harvest. Bars represent the percentage of responding cells, and error bars indicate the 95% confidence intervals determined by intercept-free linear regression analysis. Test groups are considered significantly different if the 95% confidence intervals do not overlap. (n.i.) no infection.

Since infected cells *in vivo* are not normally arrested in the IE phase, we studied the kinetics of m152 transcription in untreated cells infected with either mCMV-WT, expressing m152 only in the E phase and onward, or mCMV-m152.IE+E, expressing m152 additionally already in the IE phase (**Figure 5A**). The transcription data were then correlated with presentation of the antigenic IE1 peptide detected by sensitization of IE1-CTLL cells (**Figure 5B**). In accordance with the concept of constructing mCMV-m152.IE+E, m152 was expressed faster and inhibited antigen presentation earlier compared to infection with mCMV-WT. Specifically, at both times chosen, the IE1 peptide was not detectably presented by cells infected with mCMV-m152.IE+E, whereas it was always presented after infection with mCMV-Δm152. Under conditions of “physiological” immune evasion gene expression, as it applies to infection with mCMV-WT, the IE1 peptide was presented early in the time course but almost absent later (**Figure 5B**). We thus conclude that direct antigen presentation is largely inhibited at early and later times in cells infected with mCMV-m152.IE+E, whereas it is not inhibited at any time after infection with mCMV-Δm152, and only at later times after infection with mCMV-WT.

### Enhanced immune evasion restricts the CD8 T-cell response in BALB/c mice although increased viral replication provides more antigen for a potential cross-presentation

BALB/c mice are competent in both antigen presentation pathways and therefore theoretically have the choice between direct antigen presentation and antigen cross-presentation. This raised the question of which pathway is used as the canonical pathway for priming of naïve CD8 T cells and for subsequent clonal expansion or whether the pathways can replace each other in case of need. Assuming that direct antigen presentation is the preferred mode of priming, one expects a response magnitude in a rank order defined by the quantity of pMHC-I complexes on the surface of infected APCs and thus reciprocal to the strength of immune evasion. Specifically, the response should be strongest after infection with mCMV-Δm152, intermediate after infection with mCMV-WT, and lowest after infection with mCMV-m152.IE+E.

**Figure 6:**
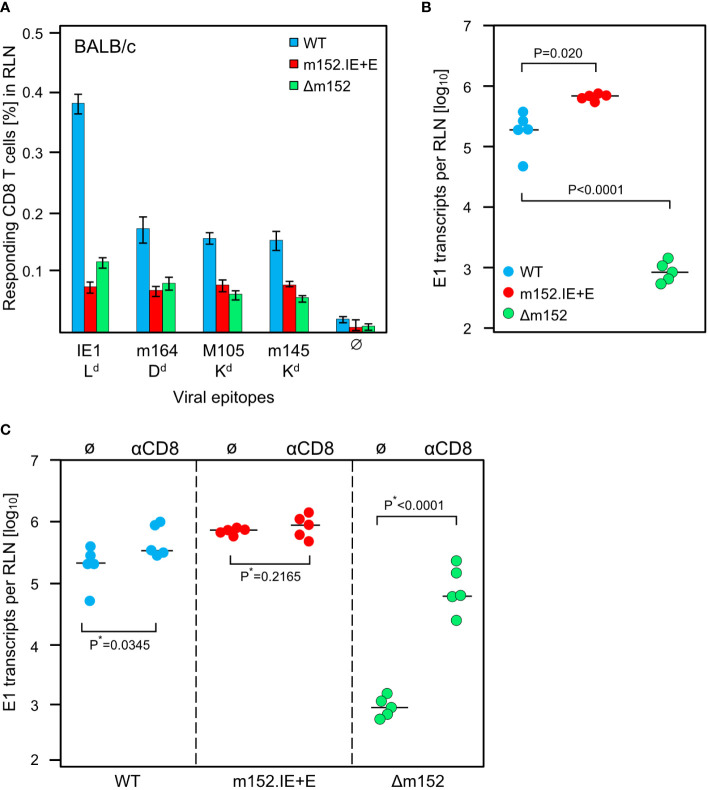
Impact of the strength of viral immune evasion on CD8 T-cell response magnitude and control of intranodal viral replication. Intraplantar infection of BALB/c mice (n=5 per group) was performed with 1x10^5^ PFU each of either mCMV-WT (WT) or mCMV-m152.IE+E (m152.IE+E) or mCMV-Δm152 (Δm152). **(A)** CD8 T-cell response in the draining RLN on day 7 post-infection. Responding CD8 T cells were quantitated in an IFNγ-based ELISpot assay, with P815 cells used as APCs that were exogenously-loaded with the indicated synthetic peptides. Bars represent cohort average frequencies of epitope-specific CD8 T cells determined by intercept-free linear regression analysis, error bars indicate the 95% confidence intervals. Test groups are considered significantly different if the confidence intervals do not overlap. Ø, no peptide added. **(B)** Viral replication in the RLN on day 3 post-infection, determined by quantitating spliced E1 transcripts by RT-qPCR. Dots represent data for individual mice. Median values are indicated by horizontal bars. **(C)** Intranodal viral replication in mice depleted of CD8 T cells on the day before intraplantar infection (αCD8) or in mice left undepleted under otherwise identical conditions (Ø). Note that data for undepleted mice are the same as in **(B)** and are shown again to facilitate the comparison with results from mice depleted of CD8 T cells. P*-values are calculated from log-transformed data using Welch´s unpaired t-test (*one-sided).

The results of the experiment (**Figure 6A**) did not match this prediction. For all antigenic peptides tested, the magnitude of the CD8 T-cell response was essentially the same for the extremes of lowest and highest direct antigen presentation after infection with mCMV-m152.IE+E and mCMV-Δm152, respectively (**Figure 6A**). It seemed to be an obvious conclusion that direct antigen presentation is not the mode of priming. However, the results for mCMV-WT do not fit this interpretation, as the CD8 T-cell response to this virus was higher compared to the two immune evasion virus mutants for the epitope panel tested (**Figure 6A**, **Supplementary Figure 1**) and was broader in terms of the epitope specificity repertoire determined by using a viral genome-wide ORF expression library (**Supplementary Figure 1**). So, if direct presentation plays no role at all, as the two antipodal mutants suggest, why is the response after infection with mCMV-WT the best, with immune evasion and direct antigen presentation being in between?

Based on our experience with the C57BL/6 model (**Figures 2**, **3**) and our published previous work in the BALB/c model (75), we quantitated replication of the three viruses in the draining RLN, the site where priming and clonal expansion take place, on day 3 after infection (**Figure 6B**). As an important control, replication differences caused by genetically-determined viral replicative fitness were excluded by showing identical replication of the three viruses in immune-deficient mice (**Supplementary Figure 2**). Therefore, replication differences in the RLN must reflect differences in immune control. Notably, unlike the sizes of the CD8 T-cell responses, the intranodal viral replication fulfilled the logic, that is, highest replication corresponds to strongest immune evasion of mCMV-m152.IE+E, lowest replication corresponds to weakest immune evasion of mCMV-Δm152, and intermediate replication corresponds to intermediate immune evasion of mCMV-WT (**Figure 6B**).

A possible contribution of recently primed virus-specific CD8 T cells to the control of intranodal virus replication was tested by CD8 T-cell depletion prior to infection (**Figure 6C**). It may be instructive that our previous work has already shown the presence of antiviral effector CD8 T cells in the RLN after 3 days of infection (75). In accordance with almost missing antigen presentation on cells infected with mCMV-m152.IE+E, intranodal viral replication was not detectably controlled by CD8 T cells, whereas, in accordance with optimal antigen presentation on cells infected with mCMV-Δm152, intranodal viral replication was almost prevented when CD8 T cells were present. Again, results for mCMV-WT were in between.

In summary, up to this point, the diametrically different levels of intranodal viral replication of mCMV-m152.IE+E and mCMV-Δm152 in BALB/c mice reflect missing and strong antiviral control, respectively, by the just generated effector CD8 T cells. Surprisingly, the result is almost the same low level of CD8 T-cell response, while the intermediate level of immune evasion by mCMV-WT results in the best response, generating high numbers of antiviral CD8 T cells exported from the RLN for controlling viral replication at distant sites of viral pathogenesis.

**Figure 7:**
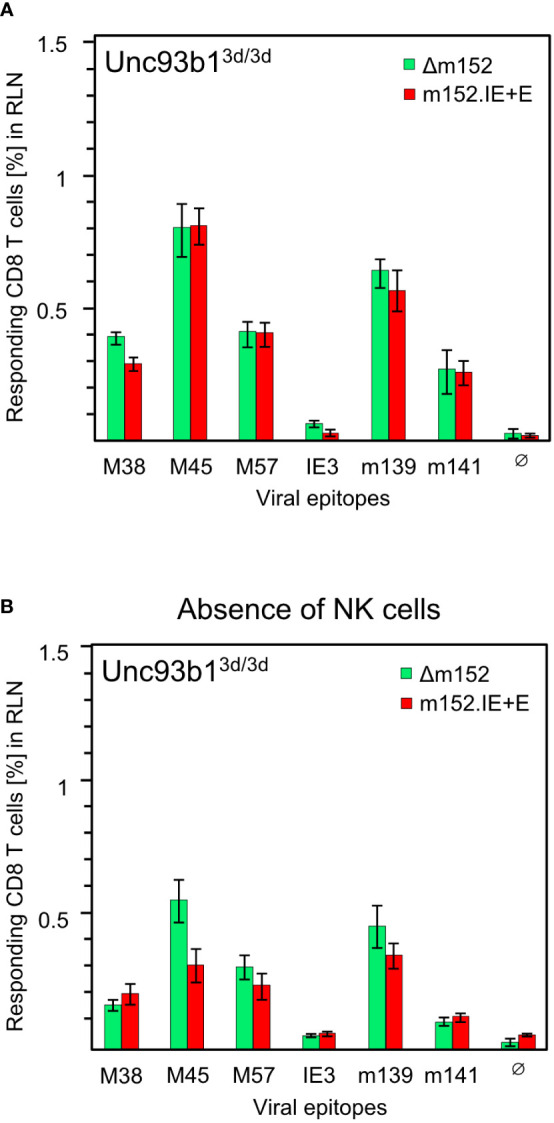
CD8 T-cell responses in absence of antigen cross-presentation. Equivalence of responses after high and low direct antigen presentation in presence **(A)** and in absence **(B)** of NK cells. CD8 T-cell responses were measured in Unc93b1^3d/3d^ mice (n=4 per experimental group) that are deficient in antigen cross-presentation. CD8 T cells were isolated from the draining regional lymph node (RLN), that is, the popliteal lymph node, on day 7 after intraplantar infection with 1x10^5^ PFU each of either mCMV-Δm152 (Δm152) or mCMV-m152.IE+E (m152.IE+E), and were used as effector cells in an IFNγ-based ELISpot assay. **(A)** No depletion of NK cells. Data are from the experiment of **Figure 1B**, where only the comparison between mCMV-WT and mCMV-Δm152 is shown. **(B)** Depletion of pan-NK cells one day before infection. For further details, see the Legend to **Figure 1**. Note that **(A, B)** represent independent experiments, so that response magnitudes can be compared reliably only between the two viruses in either experiment.

Before arriving at a conclusion, it was important to consider the possibility that direct antigen presentation and antigen cross-presentation may not be mutually exclusive. An alternative explanation for the comparable magnitude of response following high and low direct antigen presentation could be that the response to mCMV-Δm152 is actually driven by high direct antigen presentation, whereas the response to the super-evasion virus mCMV-m152.IE+E may be due to antigen cross-presentation being used as an alternative pathway, aided by large amounts of antigenic material derived from many dying cells. This tempting idea, though, was refuted by comparing the response to the two viruses in the cross-presentation deficient Unc93b1^3d/3d^ mice, revealing no notable difference for the panel of viral epitopes tested (**Figure 7A**).

As m152 also impacts on the NK-cell response by downmodulating ligands of the activatory NK-cell receptor NKG2D (47–51), high and low NK-cell responses to mCMV-Δm152 and mCMV-m152.IE+E, respectively, could indirectly affect the CD8 T-cell responses to these two viruses differentially. However, except for a minor difference in the case of viral epitope M45, the magnitude of the CD8 T-cell responses to the two extremes of immune evasion remained comparable after pan-NK cell depletion under conditions of absent antigen-cross presentation in Unc93b1^3d/3d^ mice (**Figure 7B**). As a consequence, all our results in both C57BL/6 and BALB/c mice must be explained on the basis of direct antigen presentation.

## Discussion

CMVs are often discussed as being masters in evading innate and adaptive immune control (19, 58, 103), whereas host counter-measures to ensure immune surveillance of CMV were rarely considered (104). Numerous reports published over decades specifically dealt with viral proteins, so-called immunoevasins, which subvert the MHC-I pathway of direct antigen presentation to CD8 T cells [for reviews, see (105–109)]. This may have left the medical research community with the false impression that CMVs are not controlled by CD8 T cells.

This view, however, conflicts with the undisputable fact that acute CMV infections are rapidly and tightly controlled by the immune system, with CD8 T cells being identified as the main antiviral effector cells that terminate productive acute infection and surveil latent infection for preventing virus reactivation (5, 7, 32, 77, 110). Accordingly, CMVs do not harm the immunocompetent host but cause severe and often lethal, tissue-destructing organ infection in the immunocompromised host. This fundamental observation applies to humans as well as to the mouse model [reviewed in (2, 3, 6, 9)].

CMVs are host-species specific, and different CMV species share homologous genes as well as genes with analogous function, but also possess “private genes” that have evolved for adaptation to the respective host in eons of virus-host co-evolution. So, the evolutionary acquisition and maintenance of a gene involved in limiting direct viral antigen presentation to host CD8 T cells must be expected to have a benefit for both virus and host. One hypothesis is that limiting direct antigen presentation to host CD8 T cells dampens the immune response to avoid virus clearance and to allow the virus to reach cellular niches for surviving in a state of latency. Our data now support a completely new view on the role of viral immune evasion.

It was our original aim to identify the canonical pathway of viral antigen presentation to CD8 T cells, that is, to decide between direct antigen presentation and cross-presentation. Previous studies in mouse models of CMV infection have shown that mice can mount a virus-specific CD8 T-cell response as a “plan B” by either pathway when the respective other pathway is closed genetically or experimentally (57, 59–61, 111). Specifically, as also shown here, Unc93b1^3d/3d^ mice genetically deficient in cross-presentation can perfectly develop a CD8 T-cell response by direct antigen presentation. On the other hand, mice immunized with infected MHC-I-deficient cells can develop a CD8 T-cell response by cross-presentation (59). All these reports are undoubtedly correct. However, it remained open to question which pathway is used as “plan A” when both pathways are principally accessible.

To tackle the problem, we used the novel approach of keeping the immunogenetics of the host constant and, instead, to genetically modify the virus in its immune evasion potential. This was done by enhancing and reducing direct antigen presentation relative to mCMV-WT by infection with mCMV-Δm152 and the newly constructed recombinant virus mCMV-m152.IE+E, respectively. If priming of naïve virus-specific CD8 T cells and subsequent clonal expansion are by direct antigen presentation, the magnitude of the CD8 T-cell response should have been high in case of infection with mCMV-Δm152, with which direct antigen presentation is not inhibited, and low in case of infection with mCMV-m152.IE+E, with which direct antigen presentation is strongly inhibited. The result was amazing in that the CD8 T-cell response was almost identical for the two extremes of particularly high and low direct antigen presentation, suggesting that direct antigen presentation is not the mechanism of priming. What prevented us from drawing this rash conclusion was the non-fitting finding that intermediate immune evasion, and thus intermediate inhibition of direct antigen presentation, by infection with mCMV-WT led to the best CD8 T-cell response.

Our current results concerning the response magnitude after infection with mCMV-Δm152 compared to mCMV-WT reproduce our previously published finding of a reduced response after infection with an immune evasion gene deletion virus despite enhanced direct antigen presentation (75). We called this phenomenon the “immune evasion paradox” and proposed as the mechanism a reduction of the amount of antigen available for cross-presentation due to a “negative feedback control” of intranodal viral replication effected by the just recently generated CD8 effector T cells (75). Our new data obtained with the super-evasion virus mCMV-m152.IE+E contradict a mechanism that involves antigen cross-presentation, because enhanced supply with antigenic material for cross-presentation by a high level of virus production did not improve the CD8 T-cell response. Furthermore, a contribution of antigen cross-presentation is now ruled out by analogous results obtained with Unc93b1^3d/3d^ mice genetically lacking the antigen cross-presentation pathway.

An aspect that requires explanation is the puzzling result that a CD8 T-cell response also occurs under conditions in which antigen cross-presentation is genetically excluded and direct antigen presentation is largely reduced, as is the case when Unc93b1^3d/3d^ mice are infected with the mCMV-m152.IE+E super-evasion virus. As we have recently reviewed, a tiny number of pMHC-I complexes reaching the cell surface despite interference by immune evasion proteins can suffice for recognition by high-avidity CD8 T cells (44). In addition, IFNγ is known to counteract immune evasion in the MHC-I pathway of direct antigen presentation (112, 113) by promoting MHC class-I synthesis (114) and the proteasomal processing of antigenic proteins (115). Of note, CMV immune evasion is less efficient in murine (62) and human (64) macrophages that can serve as pAPCs for direct antigen presentation.

These principles apply to BALB/c mice and C57BL/6 mice with the difference that virus replication in the RLN is restricted selectively in C57BL/6 mice by Ly49H^+^ NK cells and that even in absence of NK-cell activity the negative feedback control of viral replication by the primed CD8 T cells is less pronounced in C57BL/6 mice (**Figures 2**, **3**) compared to BALB/c mice (**Figure 6C**). This difference is most obvious in the case of enhanced direct antigen presentation after infection with the immune evasion gene deletion mutant.

**Figure 8:**
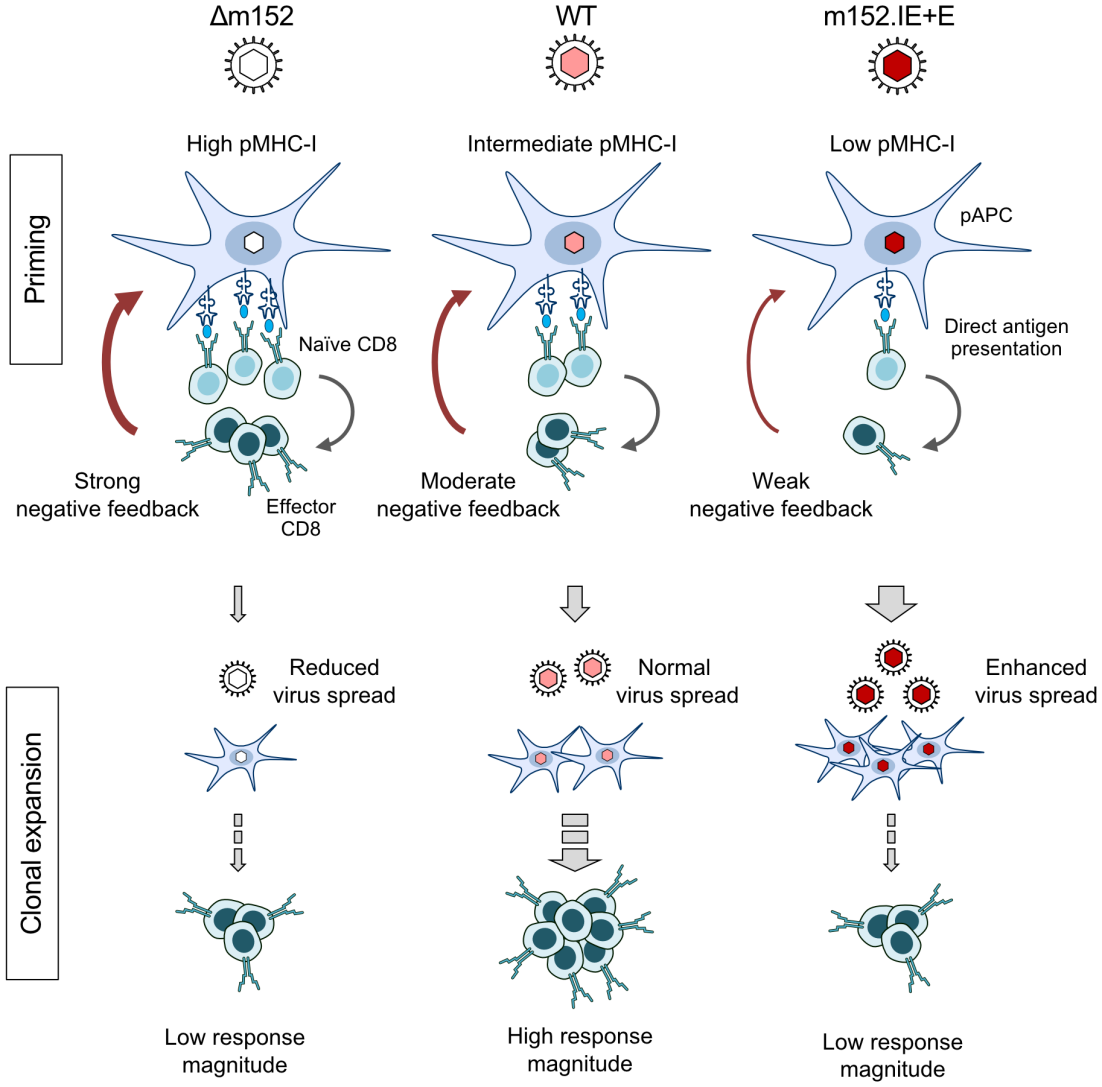
Graphical abstract. Immunoevasin-dependent CD8 T-cell response magnitude regulated by negative feedback on direct antigen presenting cells by recently primed effector CD8 T cells. (pAPC), professional antigen-presenting cells; (pMHC-I), MHC-I molecules presenting antigenic peptides. Symbols on CD8 T cells represent viral epitope-specific T-cell receptors. Growing red-color intensity symbolizes increasingly enhanced expression of immune evasion molecule m152, and thus increasing strength of immune evasion.

The negative feedback is not just a hypothesis based on our functional data, but has a structural correlate in the observation by intravital microscopy that CD8 T cells primed in the peripheral interfollicular T-cell zone of the RLN migrate back to a cortical region just underneath the subcapsular sinus, where they attack infected cells (91). The sketch in **Figure 8** summarizes the results and illustrates the proposed mechanisms. In essence, in a “negative feedback loop”, the level of direct antigen presentation during the initial priming event determines the number of primed CD8 T cells that then restrict the numbers of infected pAPCs available for driving subsequent clonal expansion.

While we have now identified direct antigen presentation as the canonical pathway for mounting a primary CD8 T-cell response in the “immunocompetent mouse model” of CMV infection in both a genetically-resistant and a genetically-susceptible mouse strain, the findings may have an even more important bearing for our understanding of viral interference with the MHC-I pathway of direct antigen presentation. We find it utmost intriguing that mCMV-WT, the virus naturally selected during virus-host co-evolution, induced the best CD8 T-cell response, whereas both prevention as well as enhancement of m152-mediated immune evasion diminished the response. It appears that optimal calibration of the strength of viral interference with the MHC-I pathway of antigen presentation serves to still allow sufficient priming of naïve CD8 T cells to initiate a response but also to moderate the “negative feedback” that otherwise would inhibit clonal expansion of the primed CD8 T cells. So, unexpectedly, viral interference with direct antigen presentation is beneficial for mounting a protective CD8 T-cell response. This sheds a completely new light on the physiological role of viral immune evasion.

## Data availability statement

The original contributions presented in the study are included in the article/**Supplementary Material**. Further inquiries can be directed to the corresponding author.

## Ethics statement

The animal study was approved by the ethics committee of the “Landesuntersuchungsamt Rheinland-Pfalz” according to German federal law §8 Abs. 1 TierSchG (animal protection law), permission number 177-07/G09-1-004. The study was conducted in accordance with the local legislation and institutional requirements.

## Author contributions

JB: Data curation, Formal analysis, Investigation, Writing – review & editing, Validation, Visualization. AF: Formal analysis, Writing – review & editing, Investigation. SB: Formal analysis, Investigation, Writing – review & editing. MB: Writing – review & editing, Conceptualization, Resources. RH: Formal analysis, Writing – review & editing, Data curation, Funding acquisition, Supervision, Validation. MR: Conceptualization, Funding acquisition, Project administration, Supervision, Writing – original draft. NL: Data curation, Formal analysis, Funding acquisition, Project administration, Supervision, Conceptualization, Validation, Visualization, Writing – original draft.

## Supplementary material

Supplementary Figure 1 Magnitude and specificity repertoire of the acute CD8 T-cell response response in BALB/c mice. Frequencies of CD8 T cells responding to infection with either mCMV-WT (WT, two upper panels) or mCMV-m152.IE+E (m152.IE+E, two lower panels) were determined by intracellular IFNγ-staining, using as stimulator cells either an mCMV genome-wide ORF library of transfectants (left panels) or P815 cells exogenously-loaded with the indicated synthetic antigenic peptides at the saturating concentration of 10^-7^ M (right panels). Responder cells were CD8 T cells isolated from the spleen on day 7 after intraplantar infection with 1x10^5^ PFU each of either of the two viruses. Note that the comparison of ORF library data for mCMV-WT and mCMV-ΔvRAP, which is equivalent to mCMV-Δm152, has been published previously (116), and supports the conclusions.

Supplementary Figure 2 Viral replicative fitness in host organs. Immunocompromised BALB/c mice (6.5 Gy of total body γ-irradiation) were infected with 1x10^5^ PFU of mCMV-WT (WT), mCMV-m152.IE+E (m152IE+E) or mCMV-Δm152 (Δm152). Viral replicative fitness was assessed by the viral doubling times (vDT), measured by M55/gB-specific qPCR in total DNA extracted from the organs indicated. Symbols represent individual mice. vDT values and their 95% confidence intervals were calculated from log-linear regression lines with ordinate intercept, including all data collected over the entire time course. Dashed curves border the respective 95% confidence areas.
