# Supplementary figures and images for: Direct antigen presentation is the canonical pathway of cytomegalovirus CD8 T-cell priming regulated by balanced immune evasion ensuring a strong antiviral response
Source: Front Immunol. 2023 Dec 12;14:1272166. doi: 10.3389/fimmu.2023.1272166 (PMC10749961; doi:10.3389/fimmu.2023.1272166)

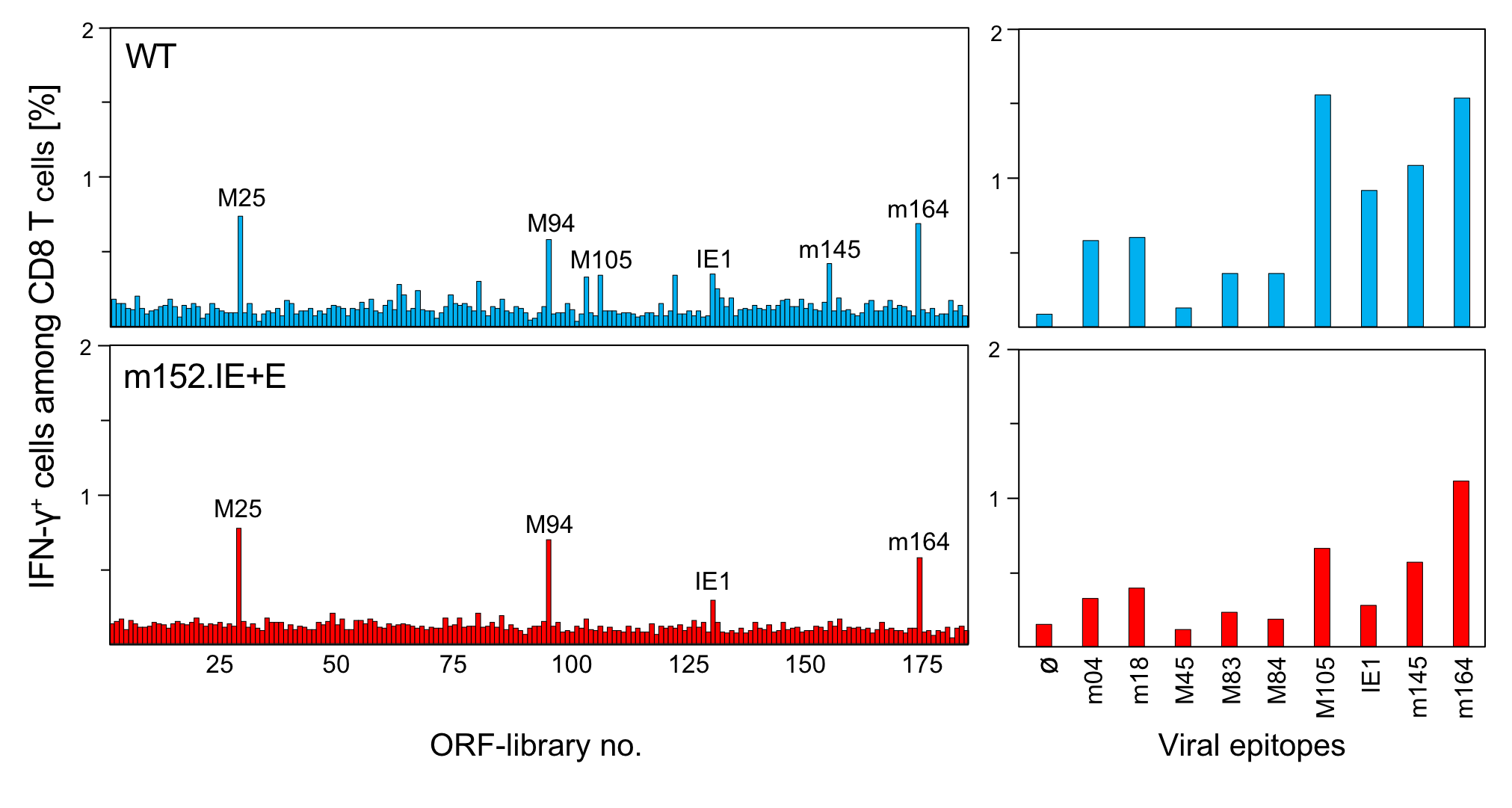

Supplement: Supplementary Figure 1 — Magnitude and specificity repertoire of the acute CD8 T-cell response response in BALB/c mice. Frequencies of CD8 T cells responding to infection with either mCMV-WT (WT, two upper panels) or mCMV-m152.IE+E (m152.IE+E, two lower panels) were determined by intracellular IFNγ-staining, using as stimulator cells either an mCMV genome-wide ORF library of transfectants (left panels) or P815 cells exogenously-loaded with the indicated synthetic antigenic peptides at the saturating concentration of 10-7 M (right panels). Responder cells were CD8 T cells isolated from the spleen on day 7 after intraplantar infection with 1x10^5^ PFU each of either of the two viruses. Note that the comparison of ORF library data for mCMV-WT and mCMV-ΔvRAP, which is equivalent to mCMV-Δm152, has been published previously (116), and supports the conclusions. [file Image_1.tif]

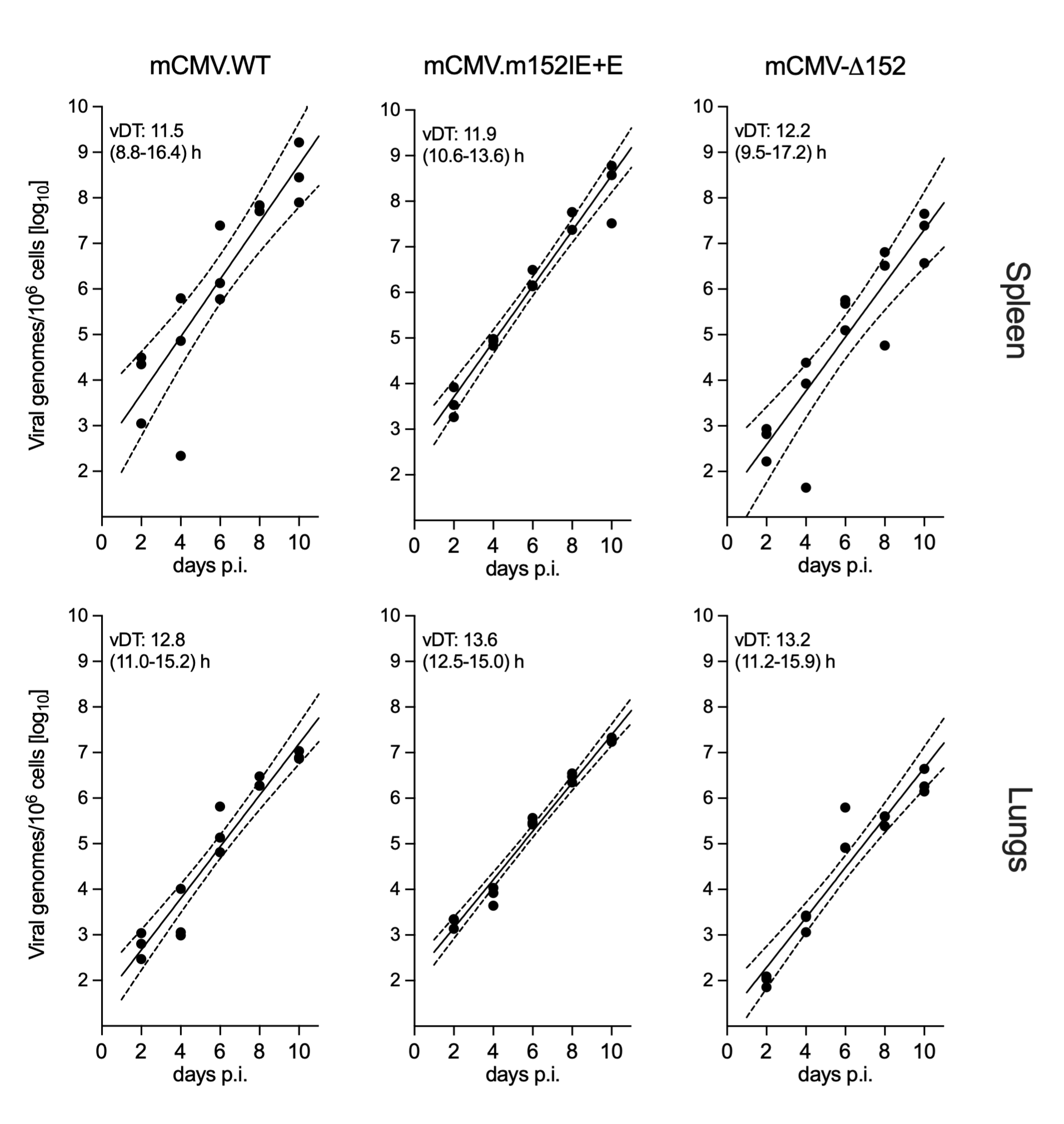

Supplement: Supplementary Figure 2 — Viral replicative fitness in host organs. Immunocompromised BALB/c mice (6.5 Gy of total body γ-irradiation) were infected with 1x10^5^ PFU of mCMV-WT (WT), mCMV-m152.IE+E (m152IE+E) or mCMV-Δm152 (Δm152). Viral replicative fitness was assessed by the viral doubling times (vDT), measured by M55/gB-specific qPCR in total DNA extracted from the organs indicated. Symbols represent individual mice. vDT values and their 95% confidence intervals were calculated from log-linear regression lines with ordinate intercept, including all data collected over the entire time course. Dashed curves border the respective 95% confidence areas. [file Image_2.tif]
